# Date-Leaf Carbon Particles for Green Enhanced Oil Recovery

**DOI:** 10.3390/nano12081245

**Published:** 2022-04-07

**Authors:** Bashirul Haq, Md. Abdul Aziz, Dhafer Al Shehri, Nasiru Salahu Muhammed, Shaik Inayath Basha, Abbas Saeed Hakeem, Mohammed Ameen Ahmed Qasem, Mohammed Lardhi, Stefan Iglauer

**Affiliations:** 1 Department of Petroleum Engineering, King Fahd University of Petroleum and Minerals, Dhahran 31261, Saudi Arabia; alshehrida@kfupm.edu.sa (D.A.S.); g201907810@kfupm.edu.sa (N.S.M.); 2 Interdisciplinary Research Center for Hydrogen and Energy Storage, King Fahd University of Petroleum and Minerals, Dhahran 31261, Saudi Arabia; ashakeem@kfupm.edu.sa (A.S.H.); g200993710@kfupm.edu.sa (M.A.A.Q.); 3 Department of Civil and Environmental Engineering, King Fahd University of Petroleum and Minerals, Dhahran 31261, Saudi Arabia; g201407800@kfupm.edu.sa; 4 Department of Reservoir Geoscience and Engineering, IFP School, 69 Avenue Paul Doumer, 92500 Rueil-Malmaison, France; mohammed.mohsen.lardhi@gmail.com; 5 School of Engineering, Edith Cowan University, 270 Joondalup Drive, Joondalup, WA 6027, Australia; s.iglauer@ecu.edu.au

**Keywords:** date leaves, pyrolysis, ball milling, carboxylic acid functionalization, carbon nanoparticle, smart water flooding, green enhanced oil recovery (GEOR)

## Abstract

Green enhanced oil recovery (GEOR) is an environmentally friendly enhanced oil recovery (EOR) process involving the injection of green fluids to improve macroscopic and microscopic sweep efficiencies while boosting tertiary oil production. Carbon nanomaterials such as graphene, carbon nanotube (CNT), and carbon dots have gained interest for their superior ability to increase oil recovery. These particles have been successfully tested in EOR, although they are expensive and do not extend to GEOR. In addition, the application of carbon particles in the GEOR method is not well understood yet, requiring thorough documentation. The goals of this work are to develop carbon nanoparticles from biomass and explore their role in GEOR. The carbon nanoparticles were prepared from date leaves, which are inexpensive biomass, through pyrolysis and ball-milling methods. The synthesized carbon nanomaterials were characterized using the standard process. Three formulations of functionalized and non-functionalized date-leaf carbon nanoparticle (DLCNP) solutions were chosen for core floods based on phase behavior and interfacial tension (IFT) properties to examine their potential for smart water and green chemical flooding. The carboxylated DLCNP was mixed with distilled water in the first formulation to be tested for smart water flood in the sandstone core. After water flooding, this formulation recovered 9% incremental oil of the oil initially in place. In contrast, non-functionalized DLCNP formulated with (the biodegradable) surfactant alkyl polyglycoside and NaCl produced 18% more tertiary oil than the CNT. This work thus provides new green chemical agents and formulations for EOR applications so that oil can be produced more economically and sustainably.

## 1. Introduction

The oil production processes from a reservoir are grouped into three classes: primary, secondary, and tertiary [1]. In the primary stage, oil is produced due to natural drive mechanisms, for example, water, gas cap, solution gas, etc. The secondary process is launched after the weakening of natural energy. Waterflooding and pressure maintenance are common secondary recovery methods. The tertiary oil recovery, known as the enhanced oil recovery (EOR) method, is introduced when the second technique is no longer economically feasible [2,3]. The common EOR methods are chemical, gas, thermal, and others [4,5,6]. The chemical EOR method involves injecting surfactant, polymer, alkaline, and alcohol chemicals to alter interfacial tension (IFT), wettability, phase behavior, and boost oil recovery. In the gas EOR method, gases such as CO_2_, N_2_, CH_4_, and Flue gas are injected into the reservoir to reduce viscosity, IFT, increase the crude’s mobility, and improve oil recovery. This is achieved due to gas mixing with the oil which results in expansion and thus pushes the oil toward production outlets. In the case of the thermal EOR methods, the temperature of the reservoir region is raised to heat the crude oil in the formation to reduce its viscosity, vaporize part of the oil, increase the mobility of the oil, and finally boost oil recovery. Common examples of thermal processes include hot water, steam, and in situ combustions, which are suitable for heavy crude oil. 

Microbial enhanced oil recovery (MEOR) falls under other EOR methods [1]. MEOR technology is an eco-friendly enhanced oil recovery method that involves the injection of microorganisms to produce surfactant, polymer, alcohol, ketone, acids, and gas in situ, to enhance the recovery of residual oil [7,8,9,10,11]. In 2013, Haq [12] introduced an environmentally friendly oil recovery method known as green enhanced oil recovery (GEOR). GEOR is a nature-friendly EOR process that injects specific green fluids, such as surfactants, polymers, alcohols, acids, ketones, and gas (N_2_, CO_2_), which boosts macroscopic and microscopic sweep efficiencies, as a result, this then increases residual oil recovery [9,10,11]. The GEOR method is divided into two types: in situ and ex-situ [11]. MEOR falls under the in situ category, whereas green chemicals (i.e., surfactant, polymer, and alcohol), smart water, gas (carbon dioxide and nitrogen), and hybrid (water alternating gas (WAG), and (FOAM)) are grouped in the ex situ process. 

This work particularly deals with the green chemical ex situ process as it involves surfactant and smart water floods. In green surfactant flooding, the eco-friendly surfactant is injected into the reservoir to reduce interfacial tension, alter phase behavior properties, and wettability alteration to improve oil recovery whereas smart water flooding (SWF) is a developing technology that utilizes modified water chemistry in terms of salinity and composition of the ions to prepare a more suitable brine composition for a specific brine/oil/rock system to achieve better recovery. The mechanisms of SWF are fine migration, pH increase, multi-ion exchange, salting-in, and wettability alteration. 

In the last decade, nanoparticles have received several applications ranging from emulsion stability [13,14] and EOR [15,16,17]. Particularly, carbon nanoparticles including carbon nanotubes (CNT), single-walled CNTs, multi-walled CNTs, and carbon dots were tested mainly in the laboratory for EOR potential. Recently, there was one test conducted in the field. While these carbon-based nanoparticles are promising, they are expensive, thus, making field applications uneconomical. As a result, the development of a cost-effective and environmentally friendly carbon nanomaterial is highly desirable. So far, date-leaf carbon nanoparticle (DLCNP) application does not extend to GEOR. This work, therefore, aims to develop carbon nanomaterial from the date-leaf via ball milling and the pyrolysis technique (the different methods of preparation are described in detail in Section 2.2) and examines its potential in GEOR. The objectives are achieved through experimental processes.

## 2. Background of the Study

### 2.1. Carbon Nanoparticle in EOR

The influence of MWCNT on IFT and surface tension was examined at room temperature by Soleimani et al. [18]. The optimum MWCNT concentration was achieved at 0.3 wt%. This solution produced 18.57% incremental oil from a glass bead experiment. The rheological properties of a mixture of an acrylamide polymer and MWCNT were tested in a high-pressure high-temperature (HP-HT) and high salinity environment [19]. Improvements in viscosity and stability in the harsh HP-HT environment were achieved by negative polyelectrolyte and polyampholytic polymers. The dispersion effects of CNT hybrids in foam and emulsion were studied in porous media by Kadhum et al. [20]. It was found that a stable CNT dispersion was obtained using a highly polarized polymer such as Arabic gum and polyvinyl pyrrolidone. An experimental study was conducted to examine the foam stability and viscosity of a surfactant polymer and MWCNT blend [21]. Investigation reveals that MWCNT could improve flow behavior in the foam of porous media. A-Dots or Arab-D dots were applied in a giant Ghawar field in Saudi Arabia to explore EOR potential [22]. A core flood experiment was conducted at 95 °C before the field trial and was followed by a post-flood with 120,000 ppm salinity brine. The average porosity, permeability, and pore volume values of the core plug were 20.3%, 9.89 mD, and 18.74%, respectively. A concentration of 0.001% *w*/*w* (10 ppm) of A-Dots solution was injected at a rate of 0.10 cm^3^/min. The solution occupied about 20% of the total pore volume (3.8 cm^3^). The oil recovery factor reported was 96%. A huff-and-puff method was applied in an Arab-D field trial. The production period was two days and the shut-in time was three days. The distance between the injection and production wells was 1 to 3 km. A total of 5 kg of A-Dot particles were mixed with 255 bbl of injected water. The solution was then injected at a rate 3300 bbl/day. The injection pressure and temperature were 1500 psi and 90 °C, respectively. The overall field trial outcome reported an oil recovery of approximately 82% implying that nano agent concepts are promising in boosting the recovery amount of trapped oil. Table 1 is a brief highlight of some nanomaterials with their EOR potentials.

### 2.2. Nanoparticle Preparation

Carbon-based nanomaterials (CBNs) are emerging as an essential topic in the fields of science and technology. Carbon and its allotropes have been used widely in various applications (such as fiber optics) due to unique aspects such as its excellent physical, chemical, thermal, electrical, and biological properties [28]. Other applications include electrochemical sensors [29,30], electronics [31], drug delivery [32], energy storage [33], solar cells [34], environmental pollutant removal [35], construction materials [36,37], and various materials science applications [38,39,40,41,42,43,44,45]. While these nanomaterials are receiving significant attention, the conventional preparation methods for CBNs are complicated and expensive, thus, limiting their utilization. Consequently, alternative forms of developing CBNs via relatively simple, cost-effective, and sustainable approaches are of great interest. CBN production from biomass could offer an ideal economic and sustainable system. The leaves from trees and other forestry are abundantly available and often go unused. It would be perfect to utilize this biological waste as a cheap material for conversion into value-added carbon products useful in several potential applications. 

Ball milling and pyrolysis, among various methods, were adopted for nanomaterial preparation and carbonization respectively. Ball milling is a simple and economical method that allows for the synthesis of nanomaterials on a large scale. It is a top-down technique where any powdered material is mechanically milled into nanoparticles using balls of various stiffnesses. The kinetics of milling depends on the milling energy, type, and size of the balls, milling speed, temperature, and duration of the milling process. Various nanocrystalline/amorphous materials were synthesized using this methodology [46,47,48,49]. On the other hand, pyrolysis is a simple and popular controlled thermochemical treatment technique that is employed to convert waste or any other biomass into valuable products. It is commonly used to prepare biochar, charcoal, and biogas for various commercial applications. Many waste materials, such as rice husk [50], jute sticks [35], date palm [51], wood waste [52], and tree/plant leaves [53,54], were converted into value-added products using this technique.

## 3. Materials and Methods

### 3.1. Materials

The date leaves were collected from the date gardens of the King Fahd University of Petroleum and Minerals (KFUPM) campus. Concentrated H_2_SO_4_ and concentrated HNO_3_ were obtained from Sigma-Aldrich. Deionized water was used in the experiments. Other materials used in this work are: (1) 99.9% pure sodium chloride (NaCl), (2) distilled water, and (3) chemical dissolvent: naphtha, ethel-methyl ketone, and acetone. 

### 3.2. Preparation of the Date-Leaf Carbon Nanoparticles and Carboxylic Acid Functionalization

**Figure 1:**
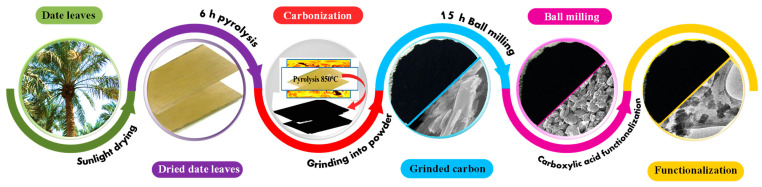
Schematic representation of the preparation of the functionalized nanoparticle date carbon.

Date leaves collected from the date gardens on the KFUPM campus served as the source of raw material. After collection, they were dried entirely in the sunlight and then cleaned with distilled water and oven-dried at 110 °C for 24 h to remove any moisture content. The leaves were then shredded into pieces 2 to 3 cm long and pyrolyzed in a tube furnace under N_2_ atmosphere at 850 °C for approximately 3 h at heating and cooling rates of 10 °C and 5 °C, respectively. The pyrolytic carbon from the dried date leaves was then powdered using a kitchen blender for approximately 5 min. (This carbon powder is referred to as “ground carbon” in the remainder of the manuscript). Next, the ground carbon was subjected to a high-energy ball-milling technique to synthesize the nanoparticles. The ball-milling machine was operated at a speed of 3000 rpm for approximately 15 h. The particle size reduction was monitored at different intervals of 3 h, 9 h, and 15 h using microscopic analysis. Zirconium balls 600 to 800 µm in size at a ratio of 1:20 by mass of Zr to carbon content were used during the ball-milling process. After completing the ball milling, the surface of the nanocarbon was modified via carboxylic functionalization with sulfuric (H_2_SO_4_) and nitric acid (HNO_3_) as per previous studies [35,55]. Typically, 1 g of the carbon particles (obtained after 15 h of ball milling) was added to a mixed acid solution (1 L of concentrated H_2_SO_4_ and concentrated HNO_3_) at a ratio of 3:1 by volume. The mixture was ultrasonicated for 5 h to form the carboxylic group. The sonicated solution was then diluted with deionized water and kept undisturbed for 8 h to settle the carboxylated carbon mass on the bottom of the beaker. The mother liquid segregated at the top was decanted. This procedure was repeated six times. Finally, the carboxylic-functionalized carbon was filtered and dried for 24 h at 60 °C. The preparation process for the functionalized date carbon is shown schematically in Figure 1.

### 3.3. Characterization

The morphologies of the ground carbon, 15 h ball-milled carbon, and carboxylic acid-functionalized carbon were characterized by field emission scanning electron microscopy (FE-SEM) (Tescan Lyra-3, Kohoutovice, Czech Republic) and transmission electron microscopy (TEM) (JEM-2011; JEOL, Tokyo, Japan). Elemental analysis was carried out using energy-dispersive X-ray spectroscopy (the Lyra-3 attachment to the FE-SEM through the LINK INCA program, Oxford, UK). Surface area, pore size distribution, and structural information were obtained using BET and Raman spectroscopic analysis. A micro-focusing X-ray monochromator XPS (ESCALAB 250Xi XPS Microprobe, Thermo Scientific, Waltham, MA, USA) was used for the X-ray photoelectron spectroscopy (XPS) analysis. All analyses were performed at the Research Institute of KFUPM in Saudi Arabia.

### 3.4. IFT Measurement

The force per unit length at the fluid–fluid and rock–fluid interfaces is referred to as interfacial tension (IFT). IFT between two fluids represents the amount of work required to create a new unit of surface area at the interface. It can also be assumed as a measure of the immiscibility of two fluids. IFT values between the Arabian light crude oil and the date-leaf solutions were measured according to standard ASTM D971-99a at different mixing ratios. Five samples of DLCNP were prepared at 200 mg/L to 800 mg/L concentration with deionized distilled water. Arabian light crude oil (*ρ*_@ 25°C_ = 0.8286 g/cm^3^) was obtained from the Saudi Aramco company. The IFT values were then measured via the ASTM D 971-99a method [56]. The step-by-step process is presented here. First, the glass containers for the experiment were cleaned using naphtha, ethel-methyl ketone, and an acetone chemical solvent. After rinsing with tap and distilled water, the glass containers were dried and covered using aluminum foil to keep them safe and clean. Second, naphtha, ethel-methyl ketone, and an acetone chemical dissolvent were then used to clean the Du Noüy ring. A butane gas flame was used as a flaming agent for approximately 5 s until the equipment slightly glowed orange. The ring dimensions are given in Table A1 in Appendix A. Third, at an ambient temperature of 25 °C, the densities of the crude oil and samples were measured to ascertain the density difference between the two liquid phases, Table A2 in Appendix A. Fourth, the heating system was then connected to the tensiometer and set to the temperature mentioned above. Fifth, intermittently during the experiment, the glass containers were cleaned and the tensiometer calibrated to avoid an error. This was achieved by ensuring that the surface tension of the distilled water measured at 25 °C fell within 71 to 73 dyne/cm. Any value outside this range signified an error from the calibration or glass. Sixth, each sample was poured into a clean glass container to a depth of 10 to 15 mm. After waiting for approximately 12 min, the temperature stabilized at 25 °C. Seventh, the ring was immersed in the sample such that its depth was less than 6 mm. Then, the heavy phase was carefully covered with crude oil such that its center was more than 10 mm. Eighth, after aging the sample-oil interface for approximately 30 s, the rupture value was recorded by lowering the platform. Finally, the IFT was then corrected through the software built into the Sigma 702 force tensiometer. The Zuidema and Waters method was used to correct the IFT value [56].

### 3.5. Contact Angle Measurement

The tendency for a solid to prefer one fluid over another is called wettability. Wettability is a function of chemical composition and it has been largely attributed to changes in the pore system (adsorption), which may be due to rock fluid interaction, fluid–fluid interactions, rock mineralogy, and brine chemistry [57]. The surface can be either oil-wet or water-wet, depending on the chemical composition of the fluids. The wettability can be determined through contact angle measurement. The contact angle can be defined as the angle formed by the intersection of the liquid–solid interface and the liquid–vapor interface. A contact angle in the range of 0–70° indicates water-wet conditions, a contact angle in the range of 70–100° indicates intermediate-wet conditions and a contact angle in the range of 105–180° indicates oil-wet conditions [57]. The Drop Shape Analyzer (DSA) 100 is designed to measure contact angle if one fluid is transparent because it measures the drop shape. The 800 mg/L (ppm) DLCNP solution and Arab light crude were selected for measuring the contact angle. The drop was made by DLCNP, and the surrounding phase was Arab Light crude oil. Both phases were dark black, and the image was not captured. Due to this difficulty, the contact angle measurement was not successful. It could be possible if a low concentration such as 100 (mg/L) was taken. However, the 100 mg/L concentration was not selected for the core flood which is vital for EOR performance prediction.

### 3.6. Influence of Pressure and Temperature on IFT

The effect of temperature and pressure on the DLCNP was determined by DSA 100. A pictorial representation of DSA 100 is shown in Figure S1 in the Supplementary Material. First, N-Hexane was used to clean the IFT cells, lines, and cylinder before applying acetone and water. The equipment was then dried with an airflow. Second, the IFT of water at 72.2 mN/m was used to calibrate the IFT cell under ambient conditions. The single-piston syringe pump and/or hand pump of the automated HP were used to fill both cells by transferring the relevant and required fluids. Third, the IFT cell was filled with brine using the hand pump before calibrating the system. The system calibration was performed by measuring the needle size while the software was running. Then, an oil bubble was formed by pumping crude oil through the needle. The injection of brine set the test pressure into the cell, and a thermocouple was used to set the temperature surrounding the cell. Finally, after the desired pressure and temperature were attained for the measurement, the cell was kept for approximately 30 min under stable conditions to allow the two phases to reach equilibrium. At this point, the IFT was measured by actively running the software.

### 3.7. Phase Behavior Study

The phase behavior of a green surfactant system is an important parameter for the oil recovery process. The aims were to determine the middle phase microemulsion and optimum salinity of the green surfactant system. The phase behavior is a simple laboratory technique utilizing the salinity concept. The surfactant concentration was kept constant, NaCl concentration was varied, and the water–oil ratio (WOR) was 1. The volume of oil was 4.5 mL, and the volume of the aqueous phase was 4.5 mL. The aqueous phase consisted of 0.50% APG surfactant and varied (1–7%) NaCl concentrations. The stable phase volume was measured when it reached equilibrium. The calculated volumes are given in Table 2 with an error limit of ±0.01.

### 3.8. Core Flood Experiments

Core flood experiments were performed to determine the behavior of nanofluid inside the reservoir at reservoir pressure and temperature levels. The amount of oil recovered by a given formulation during a core flood can provide a good indication of the possible tertiary recovery potential under real reservoir conditions. Initially, the core samples after cutting and oven-dried (50 °C) for 3 days were measured and placed in the core holder where an overburden pressure (1050 psi) was applied. Thereafter, a vacuum was applied to the core for 24 h to remove any air. The core was saturated with a brine of 2% NaCl concentration and then flooded with 30° API Arab light crude oil (drainage) until the core reached residual water saturation ($S_{wi}$); at this point, the core was saturated with oil. Then imbibition process of the oil-saturated core sample began by flooding with 2% NaCl brine at an injection rate of 0.50 cm^3^/min until it was near residual oil saturation (Sor) and ready for the formulations (nanofluid/surfactants) treatment. Typically, brine flooding was stopped when only a trace of oil was being produced. The next step was to continue injection by the different formulations at 0.50 cm^3^/min. Post flooding was carried out thereafter, to ensure that no oil remains in the tube and cores. The schematics for the core flood experiment setup are presented in Figure S2 in the Supplementary Material. The properties of the core samples used in the experiment are presented in Table 3 whereas the formulation of the three nanoparticle/surfactant combinations is given in Table 4.

## 4. Results and Discussion

### 4.1. Field Emission Scanning Electron Microscopy

**Figure 2:**
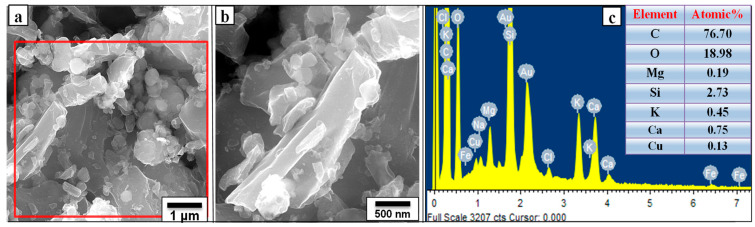
(**a**,**b**) FE-SEM images of the ground carbon at two different magnifications; (**c**) the EDS spectra recorded at the red marked area indicated in the FESEM image (**a**).

Figure 2a,b are the FE-SEM images of the ground carbon at two different magnifications. The corresponding elemental analysis is shown in Figure 2c. From Figure 2a,b, it is clear that the particle sizes of the carbon powder were widely distributed, typically ranging from 0.5 µm to 6 µm. The elemental analysis of the ground carbon indicated that C and O existed in significant quantities, while only traces of Si, Ca, K and Mg were present. Note that Cu and Au peaks were caused by the copper substrate and gold coating on the surface of the sample.

**Figure 3:**
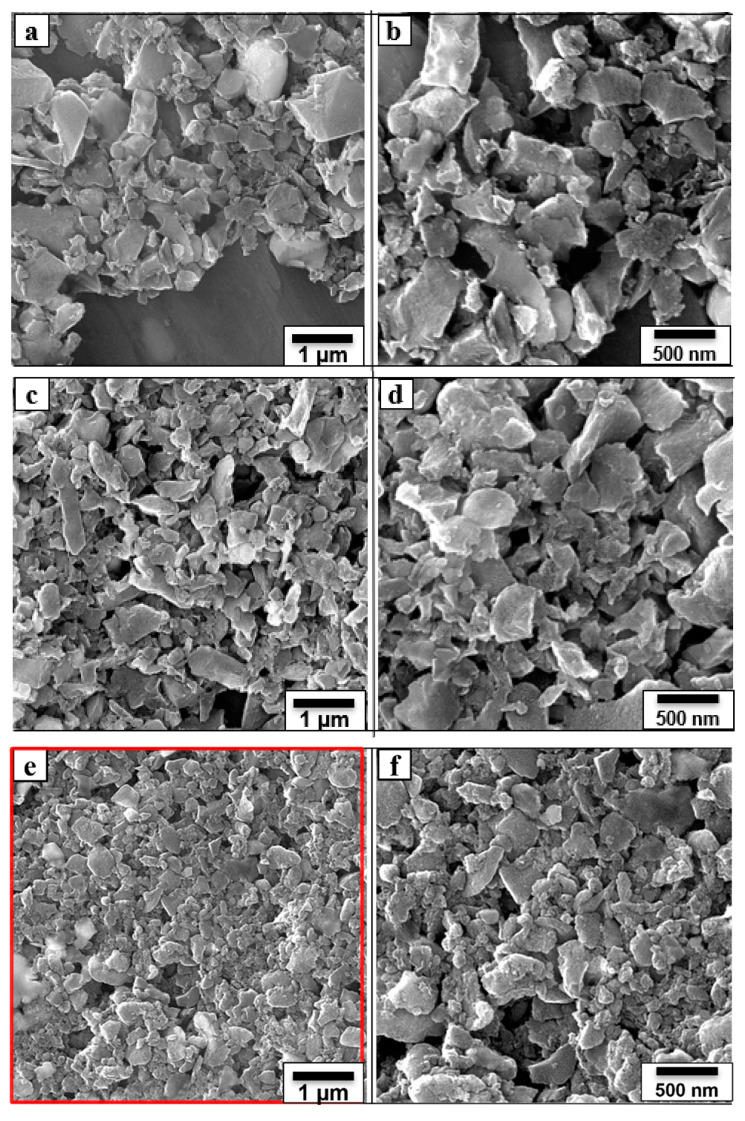

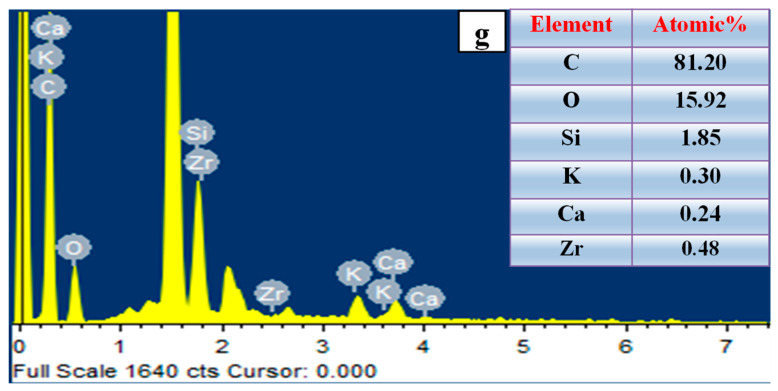
FE-SEM images of the ball-milled carbon at two magnifications (**a**,**b**) 3 h; (**c**,**d**) 9 h; (**e**,**f**) 15 h; (**g**) the EDS of the 15 h ball-milled carbon recorded at the red colored area indicated in FESEM image (**e**).

The size reduction of the carbon particles was monitored by measuring the grain size via FE-SEM imaging. Figure 3a–f show the FE-SEM images of the ball-milled carbon at three intervals (3, 9 and 15 h) on two magnification scales intended to measure the size reduction, while Figure 3g represents the chemical composition of the 15 h ball-milled carbon. Figure 3a,b are the FE-SEM images of the 3 h ball-milled carbon at two magnifications (1 µm and 500 nm). The particle size was reduced significantly by the ball milling. A typical particle size measurement on the FE-SEM images indicated particles of the following sizes: 28% > 1 µm, 60% between 1 µm and 500 nm, and 12% between 500 and 300 nm. The average particle size was around 840 nm. Figure 3c,d are the FE-SEM images of the 9 h ball-milled carbon at two magnifications (i.e., 1 µm and 500 nm). It can be observed that the particle size was further reduced by continued ball milling. Here, the particle size was 7% > 1 µm, 30% between 1 µm and 500 nm, 50% between 500 and 300 nm, and 12% between 300 and 100 nm with an average particle size of 570 nm. Similarly, 15 h ball milling, the particle size was significantly further reduced, i.e., particle sizes were 0% > 1 µm, 11% between 1 µm and 500 nm, 26% between 500 and 300 nm, 37% between 300 and 100 nm, and 26% < 100 nm, with an average particle size of 270 nm, Figure 3e,f. Hence, ball milling reduced the particle size of carbon from a few microns to the submicron/nanoscale. The EDS shown in Figure 3g indicates the elemental composition of the nanocarbon. The presence of Zr is attributable to the zirconium balls used for the ball milling.

### 4.2. Transmission Electron Microscopy

**Figure 4:**
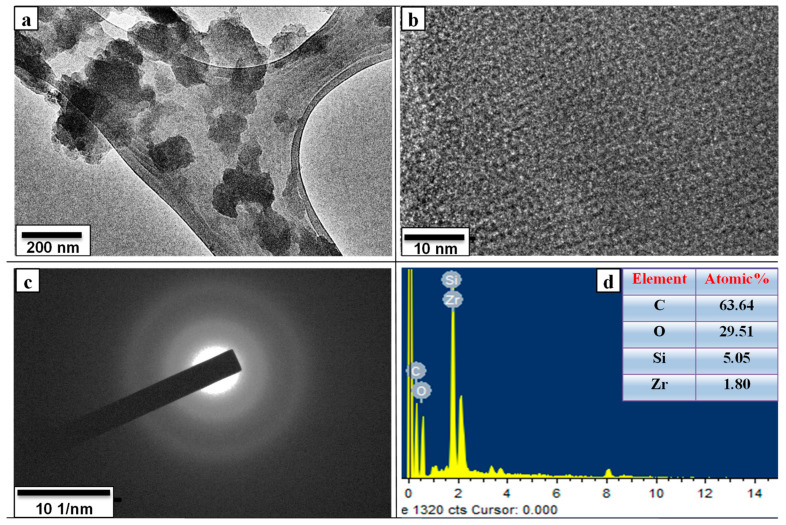
(**a**) TEM; (**b**) HRTEM micrographs; (**c**) SAED pattern; (**d**) EDS of the carboxylic functionalized nanosized date carbon.

Figure 4a is the TEM image of the nanosized date carbon after functionalization with sulfuric and nitric acid at 200 nm magnification. The particle grain boundaries on the TEM grid are visible in Figure 4a, indicating that the particle size was reduced due to the carboxylic functionalization; most of the particles shrank to the 50 to 150 nm range. The HRTEM image shown in Figure 4b indicates the amorphous nature of the carbon, as no lattice fringes can be observed. This was also confirmed from the selected area electron diffraction (SAED) pattern shown in Figure 4c. The elemental composition of the functionalized nanosized date carbon is shown in Figure 4d, indicating that C and O were the major elements while Si and Zr appeared only in trace amounts.

### 4.3. XPS Analysis

**Figure 5:**
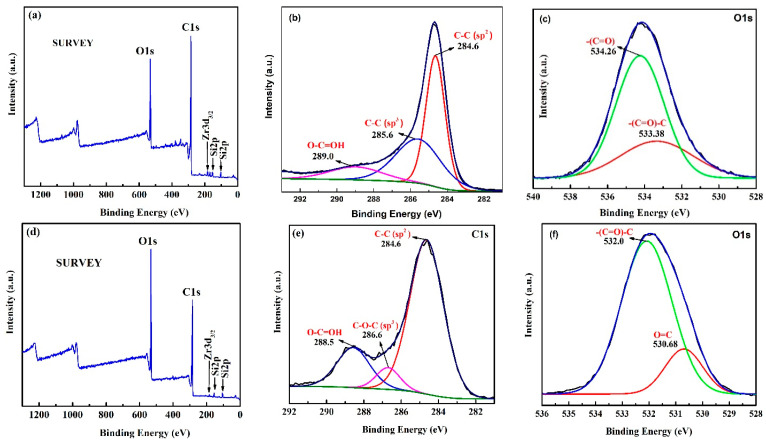
XPS spectrum of ball-milled (15 h) carbon (**a**) Survey; (**b**) C1s; (**c**) O1s; and functionalized carbon (**d**) Survey; (**e**) C1s; (**f**) O1s.

The functional group’s formation on the surface of the carboxylic functionalized carbon was studied using the XPS technique. The XPS survey and deconvoluted C1s and O1s spectrum before (i.e., 15 h ball-milled carbon) and after the acid functionalization carbon are shown in Figure 5. The XPS survey spectrum in Figure 5a,d indicates the existence of C and O as major constituents of the carbon, Si is attributed from the glass substrate/from the carbon sample as a trace amount of Si was observed in EDS analysis of the carbon. While minor traces of Zr are from the ZrO_2_ ball used during ball milling. Figure 5b,c depict the deconvoluted C1s and O1s XPS spectrum of 15 h ball-milled carbon. The C1s deconvoluted XPS spectrum indicates the presence of a C–C sp^2^ hybridized peak at 284.6 eV, C–C sp^3^ peak at 285.6 eV, and O–C=OH at 289.0 eV, respectively, while the O1s deconvoluted XPS spectrum exhibits two peaks at 534.26 eV and 533.38 eV corresponding to –(C=O) and –(C=O)–C bond, respectively [58]. The deconvoluted XPS spectrum of C1s and O1s after carboxylic acid functionalization is shown in Figure 5e,f. The functionalized carbon C1s deconvoluted spectra exhibited a C–C sp^2^ peak at 284.6 eV, C–O–C sp^3^ peak at 286.6 eV, and O–C=OH peak at 288.5 eV, respectively, while the deconvoluted O1s XPS spectrum of the functionalized carbon indicates two peaks at 532.0 eV and 530.68 eV, respectively, corresponding to –(C=O)–C and O=C bond [58]. It is noted that the peak area of the O–C=OH (Figure 5e) peak in the acid-functionalized carbon is much higher than the ball-milled carbon (Figure 5b). Thus, the XPS technique indicates the formation of carboxylic functional groups after the acid functionalization of the carbon. The functionalized group formation is similar to the study reported earlier [35,55,59]. Table 5 is a quantitative elemental analysis from the XPS survey spectrum before and after carboxylic acid functionalization of the carbon.

### 4.4. BET Analysis

**Figure 6:**
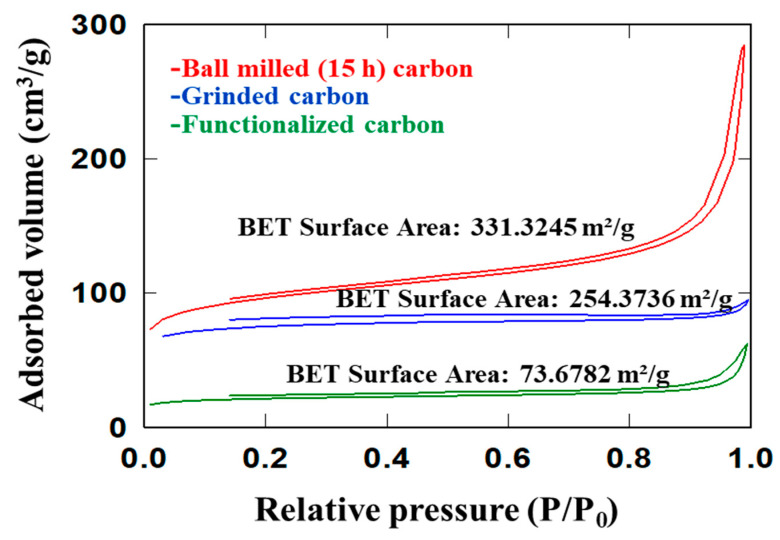
Nitrogen adsorption/desorption BET isotherms of the ground, ball-milled (15 h), and functionalized date carbon.

The specific surface area of the carbon was determined using N_2_ adsorption–desorption isotherms by the Brunauer–Emmett–Teller (BET) method [60], while the pore size distribution was analyzed using the Barrett–Joyner–Halenda (BJH) method. The N_2_ adsorption/desorption isotherms at 77 K for the ground carbon, 15 h ball-milled carbon, and 15 h ball-milled carboxylic-functionalized carbon are depicted in Figure 6. As per the IUPAC classifications, the isotherms for the ground and functionalized carbon appeared to be Type I, indicating that the size distribution of the carbon was microporous. The overlapping adsorption–desorption curves suggest that the micro-porosity of the carbon was narrow and uniform. The 15 h ball-milled carbon belonged to the Type II isotherm, with a relatively high adsorption/desorption capacity. The complete hysteresis loop between 0.4 and 1.0 (P/P_o_) indicated mesopores, while the sudden increase in adsorption from 0.9 to 1.0 (P/P_o_) demonstrated the presence of macropores [61]. The BET surface area of the ground carbon increased from 254.4 m^2^/g to 331.3 m^2^/g due to the 15 h of ball milling, while the carboxylic functionalization of the 15 h ball-milled carbon significantly decreased the BET surface area from 331.3 to 73.7 m^2^/g. This reduction in BET surface area, nearly five times that of the ball-milled carbon, can be attributed to the oxidation of the carbon with the acid. The decrease in BET surface area due to carboxylic functionalization is consistent with data reported in other studies [35,62]. 

The BJH pore size distributions of the ground, 15 h ball-milled, and functionalized carbons are shown in Figure S3 in the Supplementary Material. Figure S3a depicts the pore distribution of the ground carbon, with a pore width ranging from 1.9 nm to 230 nm, an average pore width of 2.1 nm, and a total pore volume of 0.13 cm^3^/g. The average pore width and total pore volume increased to 3.67 nm and 0.30 cm^3^/g after 15 h of ball-milling, Figure S3b. However, carboxylic functionalization of the ball-milled carbon reduced the average pore width to 3.164 nm and total pore volume to 0.058 cm^3^/g, as shown in Figure S3c. The physical properties of the date carbon at various stages of processing are tabulated in Table 6.

### 4.5. Raman Spectroscopy

**Figure 7:**
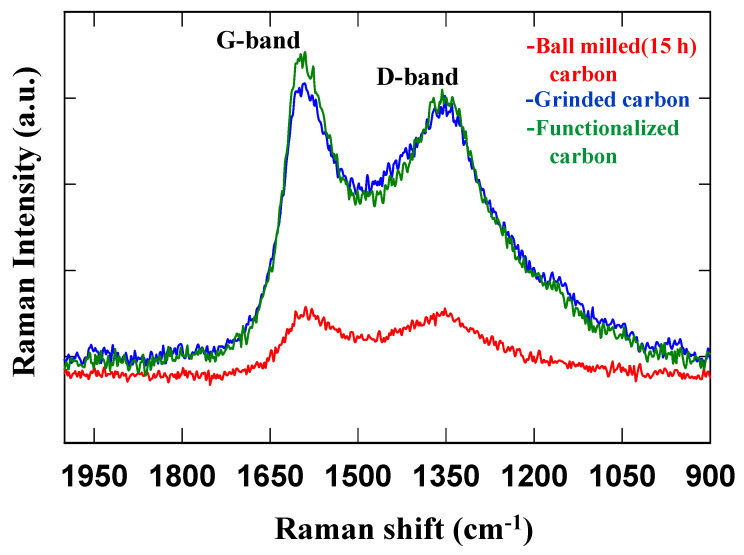
Raman spectra of the ground, 15 h ball-milled, and functionalized date carbon.

Figure 7 shows the Raman spectrum of the ground pyrolytic date carbon (pyrolysis at 850 °C) after being ball-milled for 15 h, and subsequently, acid-functionalized, measured at a wavelength of 532 nm. The two intense peaks in the spectrum correspond to graphitization (G-band) and defects (D-band). The G-band and D-band peaks for the ground carbon can be observed at around 1591 cm^−1^ and 1352 cm^−1^, respectively. For the 15 h ball-milled carbon, the G-band and D-band values were also around 1589 cm^−1^ and 1352 cm^−1^. In contrast, for the functionalized carbon, the G-band and D-band values were 1591 cm^−1^ and 1357 cm^−1^. The intensities of the G- and D-bands of the ground and functionalized carbon were almost the same, but significantly lower and broader for the 15 h ball-milled carbon. The I_D_/I_G_ ratio (I_D_ signifies the intensity of the D band, I_G_ signifies the intensity of the G band) indicates the defect sites (the degrees of disorder) for the ground carbon, ball-milled carbon, and functionalized carbon are 3.78, 2.67 and 2.74, respectively. It indicates the improvement in the ordered carbon with ball milling and functionalization.

### 4.6. IFT Measurements and Optimum Concentration at 25 °C

**Figure 8:**
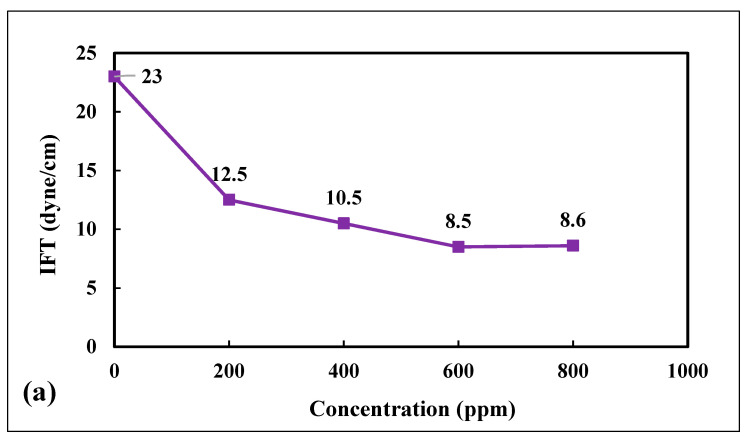

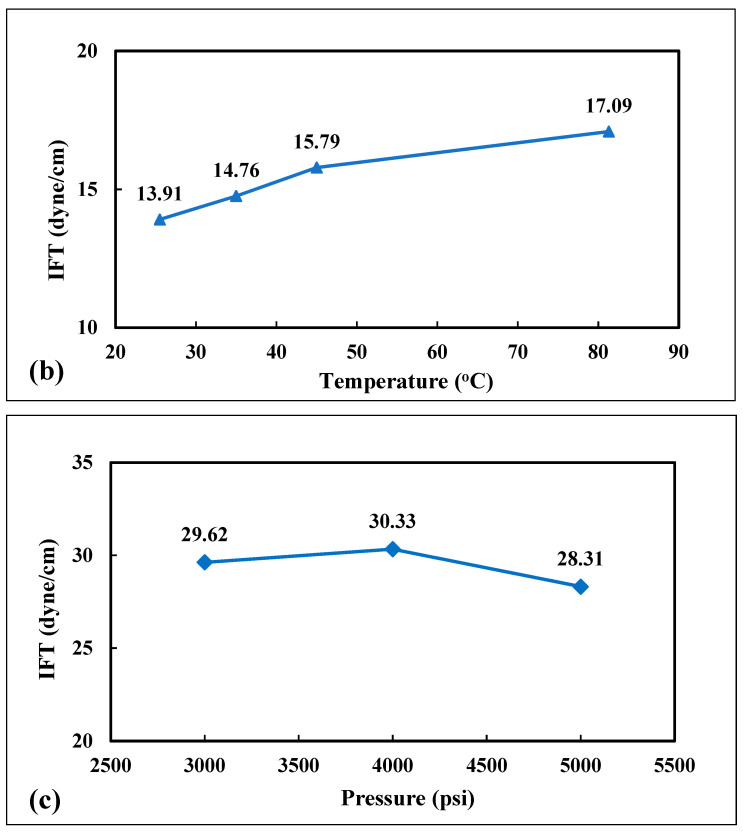
(**a**) IFT versus DLCNP concentrations for Arabian light crude oil at laboratory conditions; (**b**) Influence of temperature on IFT; and (**c**) Influence of pressure on IFT.

Five samples of DLCNP were prepared at 200 mg/L to 800 mg/L concentration with deionized distilled water. Table A3 in Appendix A presents the IFT values, and Figure 8a is a plot of the IFT values against their respective DLCNP concentrations. At a DLCNP concentration of 0.00 mg/L, the IFT was 23 dyne/cm. This value decreased to about 8.5 dyne/cm when the DLCNP concentration increased to 600 mg/L, above which it plateaued out; the optimum concentration was thus taken to be 600 mg/L. This was also consistent with our earlier study on IFT [27].

### 4.7. Wettability Measurement and Influence of Temperature and Pressure on IFT

As previously highlighted in Section 3.5, the wettability via contact angle measurement was not successful. This was attributed to the fact that since both phases were dark black, distinguishing between the drop and the surrounding phase from the image was not successful. Moreover, the DSA 100 is designed to measure contact angle if one fluid is transparent because it measures the drop shape. Furthermore, at higher concentrations, the carbon materials appeared dark, hence, measurement was unsuccessful.

The impact of temperature and pressure on IFT was observed by DSA 100 as highlighted in Figure 8. IFT was measured at temperatures ranging from 25 °C to 81 °C. It was observed that IFT increased from 14 dyne/cm to 17 dyne/cm as the temperature increased from 25 °C to 81 °C, Figure 8b and Table A4 in Appendix A. Thus, a 56-degree rise in temperature only increased IFT by 3 dyne/cm. This means that temperature had only a minimal influence on the IFT values of DLCNP solutions. Furthermore, the IFT values of the DLCNP solutions were measured with pressures varying from 3000 psi to 5000 psi. There was a 2 dyne/cm change in IFT (from 28 dyne/cm to 30 dyne/cm), Figure 8c and Table A5 in Appendix A; thus, a minimum influence of pressure on IFT from the 3000 psi to 5000 psi pressure range. The study by Soleimani et al. [23], presented in Table 1, also observed an increase in surface tension measurement (since theoretically, surface tension has an indirect relationship with IFT), between CNT and air (because IFT measurement between CNT and the Dubai crude oil sample used was unsuccessful due to a very dark cloudy phase), with a maximum value of 33.46 dyne/cm at 0.3 wt% CNT. The authors conclude that CNT synthesized via chemical vapor deposition technique can lower IFT, hence, an improvement in oil recovery can be achieved. Though the authors did not measure the effect of temperature and pressure [23], perhaps, it may be attributed to the minimal impact the CNT had as we did observe in our DLCNP as seen in Figure 8b,c.

### 4.8. Optimum Salinity of Green Surfactant

**Figure 9:**
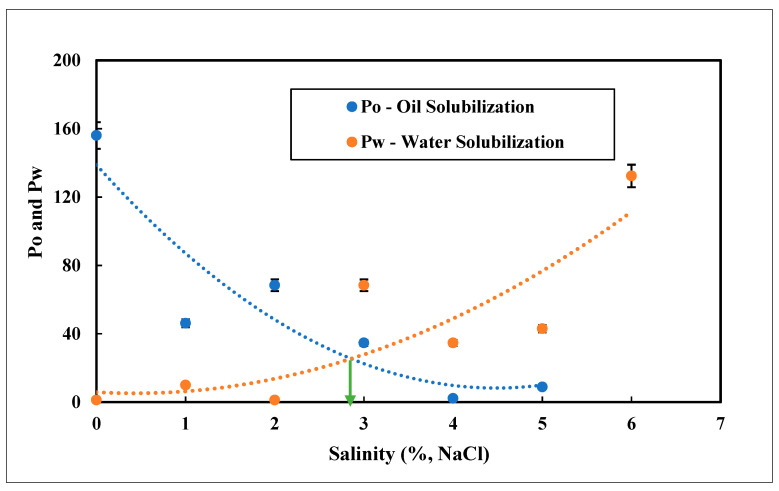
Optimum salinity of the APG—NaCl system.

The optimum salinity of the APG and NaCl system was measured through Healy and Reed correlation. The calculated data are given in Table 7. The solubilization parameters of oil and brine in the micro-emulsion phases were calculated using Equations (1) and (2). Then the parameters were plotted against salinity. The optimum salinity was obtained by the point of intersection between the oil and water curves. Figure 9 describes the optimum salinity of the APG–NaCl system where an optimum salinity of about 2.75% was observed.

Solubilization parameter of oil,

(1) $$P_{o}=\frac{V_{o}}{V_{s}}=\frac{\mathrm{Vol}.\;\mathrm{of}\;\mathrm{oil}\;\mathrm{in}\;\mathrm{microemulsion}\;\mathrm{phase}}{\mathrm{Vol}.\;\mathrm{of}\;\mathrm{surfactant}\;\mathrm{in}\;\mathrm{microemulsion}\;\mathrm{phase}}$$

Solubilization parameter of water,

(2) $$P_{w}=\frac{V_{w}}{V_{s}}=\frac{\mathrm{Vol}.\;\mathrm{of}\;\mathrm{water}\;\mathrm{in}\;\mathrm{microemulsion}\;\mathrm{phase}}{\mathrm{Vol}.\;\mathrm{of}\;\mathrm{surfactant}\;\mathrm{in}\;\mathrm{microemulsion}\;\mathrm{phase}}$$

### 4.9. Core Flooding

In this study, three core flood experiments were conducted to test the EOR capability of functionalized and non-functionalized DLCNP. The first experiment was carried out to see how functionalized DLCNP worked in smart flooding and increased the tertiary oil. The goal of the second core flood test was to observe how a mixture of non-functionalized DLCNP and non-ionic green surfactant boosted residual oil in the EOR process. The last formulation was conducted to compare the tertiary oil recovery (TOR) of the DLCNP with that of commercial CNT.

#### 4.9.1. Functionalized DLCNP in Smart Water Flooding

**Figure 10:**
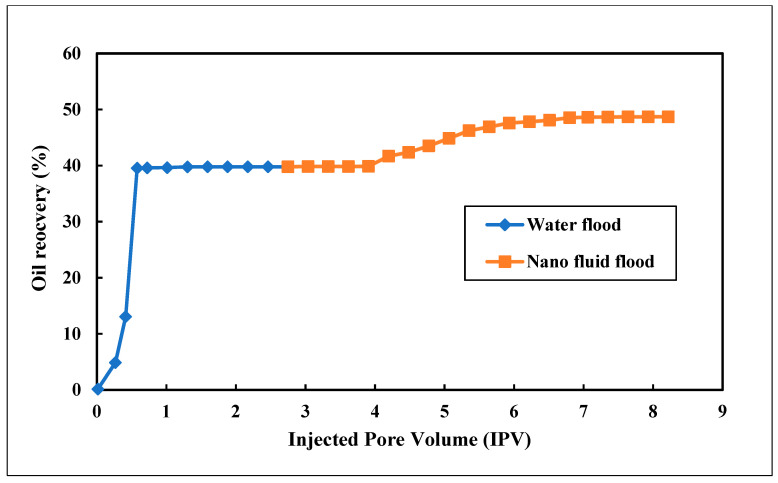
Total oil recovery observed after water and nanofluid flooding.

The impact of DLCNP and distilled water on oil recovery was studied via a core flood experiment. In the formulation reported here, 800 mg/L (ppm) of DLCNP was mixed with distilled water. After water flooding, the secondary oil recovery was 40% of the oil initially in place (OIIP). About three pore volumes of nanofluid were then injected into the core, and incremental oil recovery was 9%; thus, total oil recovery was 49% of OIIP. Figure 10 shows the complete recovery calculated after brine and DLCNP mixture flooding.

#### 4.9.2. DLCNP in Green Surfactant Flooding

The influence of non-functionalized DLCNP and non-ionic green surfactants mixture together with NaCl on oil recovery was studied in this experiment. The non-ionic surfactant APG 264 of 0.5% and non-functionalized DLCNP of 800 mg/L (ppm) were mixed with 2% NaCl. Results of this formulation are presented in Figure S4 in the Supplementary Material. In the water flooding stage, secondary oil recovery was 44% of Oil Initially in Place (OIIP). In surfactant and carbon nanomaterial flooding, there was 33% oil production after 4.6 PVs of the fluid injection and 12.33% oil production after 1.3 PVs of brine injection. Total tertiary oil recovery was 45%.

#### 4.9.3. Non-Functionalized DLCNP with CNT

A batch of (non-functionalized) DLCNP was prepared and compared with (non-functionalized) CNT. The core flood experiment was described in detail earlier [8,9,10,11]. The non-ionic surfactant APG 264 of 0.5% and CNT of 800 mg/L (ppm) were mixed with 2% NaCl. Results of this formulation are presented in Figure S5 in the Supplementary Material. The secondary oil recovery was 50% of Oil Initially in Place (OIIP). In surfactant flooding, there was 23% oil production after 3.3 PVs of nanofluid injection and 4% oil production after 0.6 PVs of brine injection. Total tertiary oil recovery was 27% [63]. A summary of the three formulation results is presented in Table 8, whereas Figures S5 and S6 represent the oil recovery observed. The efficacy of using green surfactant and co-surfactant systems for EOR was studied previously by [12]. The surfactant contained 0.5% of APG 264 and 0.5% butanol as co-surfactant. The oil recovery due to waterflood was about 41% and the tertiary oil recovery of this formulation amounted to 41%. This is a considerable amount of additional oil recovery, implying a total oil recovery of approximately 82% of OIIP. The tertiary oil recovery of APG and DLCNP (formulation II) is 45% which is about 4% more than the APG and butanol system studied by [12]. This, therefore, shows that the carbon particle might be an eco-friendly option for boosting oil production.

## 5. Conclusions

This study experimentally investigated the potential application of carbon nanomaterial for green EOR studies. Based on this research, the main findings of this paper are:

1. A carbon nanoparticle was prepared from a cheap source via a direct method (i.e., pyrolysis at 850 °C and ball milling for 15 h). The surface of the nanocarbon was carboxylic acid-functionalized using acid treatment. The prepared carbons were characterized using FE-SEM, TEM, EDS, XPS, Raman spectroscopy, and BET.
2. The particle size of the carboxylic acid-functionalized carbon ranged from 50 to 159 nm.
3. The IFT values between the Arabian light crude oil and different concentrations of aqueous DLCNP were measured. The DLCNP reduced the IFT by 41% from 14.46 to 8.56 dyne/cm at 25 °C.
4. Only a minimal influence of pressure and temperature on the IFT between the DLCNP solutions and Arabian light crude oil was observed.
5. Core flood experiments with Berea sandstone confirmed that a concentration of 800 ppm carboxylated DLCNP mixed with distilled water could recover 9% of the residual oil and 49% of the OIIP.
6. An 800 ppm sample of non-functionalized DLCNP was blended with 0.5 wt% non-ionic green surfactant APG and 2 wt% NaCl brine; this produced 45% tertiary oil and 89% OIIP recovery. This formulation performed better than the commercially available CNT.

The date tree is available all over the Kingdom of Saudi Arabia and in the middle east. The nanoparticle developed from the leaf and utilized to boost oil recovery will add value to the national economy.

## 6. Recommendations

It is recommended that the wettability study via contact angle measurement between carbon particle solution and crude oil system be investigated further. Additionally, since both fluids are black, it would be better to develop a new and effective measuring technique. Additionally, the narrow-down salinity scanning technique should be conducted to get a better understanding of the optimum salinity.

## Figures and Tables

**Table 1:** Selected publications of carbon nanotube (CNTs) application in EOR studies.

Type of CNTs	Applications	Exp. Conditions	Particle Synthesis	Particle Characterization ^1^	Key Findings	Refs.
MWCNTs	IFT reduction using pendant drop.	Ambient temperature and pressure	Chemical vapor deposition	XRD and TEM	A significant reduction in IFT was observed between the crude and CNTs at the highest surface tensions.	[23]
MWCNTs	Influence of Electromagnetic (EM) waves on nanofluids in EOR.	Ambient temperature and pressure	-	-	EM-based hybrid MWCNTs recorded optimum nanofluids flow rate of 2 mL/min. EM wave could enhance recovery efficiency by 24.5% ROIP as oil increased from 36.6% to 61.1%.	[24]
MWCNTs/Biopolymer	The effect of CNT in a harsh high-salinity high-temperature (HS-HT) environment in EOR.	High salinity and temperature	Free-radical copolymerization	NMR, GPC, FTIR, DSC and TEM.	After several syntheses, the negative polyelectrolyte and polyampholytic polymers served as the best candidate for MWCNTs/polymers in remote environments.	[19]
Magnetic iron core-carbon shell nanoparticles	IFT studies via spinning drop. Adsorption reduction studies. EOR potential.	-	Hydrothermal process	SEM, DLS, N_2_-physisorption, XRD, XRS and BET analysis.	Achieved an optimum concentration with nano-additives. Nanoparticles reduced surfactant adsorption by 33%. A final oil recovery of 98% was achieved.	[25]
Natural aluminosilicate nanomaterial halloysite nanotubes (HNTs)/SiO_2_	Wettability alteration studies. EOR potential.	Ambient pressure and temperature	-	XRD, TGA, TEM and Zeta potential measurements.	A significant change in wettability from oil-wet to water-wet. Ultimate recovery of 39% was achieved.	[26]
Date-leaf carbon micro-nano structured particles (DLCMNPs) functionalized with carboxylic acid	EOR application through IFT reduction via ring method.	Ambient temperature and pressure	Pulverization	FESEM, SEM, EDS, TEM and XRS.	IFT reduction between Arab crude oil and fluid samples formulations was achieved Nanoparticle decreased IFT from 14.46 to 8.56 dyne/cm.	[27]

^1^ XRD—X-ray Diffraction, TEM—Transmission Electron Microscopy, NMR—Nuclear Magnetic Resonance, GPC—Gel Permeation Chromatography, FTIR—Fourier Transform Infrared Spectroscopy, DSC—Differential Scanning Calorimetry, SEM—Scanning Electron Microscopy, DLS—Dynamic Light Scattering, XRS—X-ray Spectroscopy, BET—Brunauer–Emmett–Teller, TGA—Thermal Gravimetric Analysis, FESEM—Field Emission Scanning Electron Microscopy, EDS—Energy Dispersive Spectroscopy.

**Table 2:** Phase behavior experimental data.

Salinity	Initial Vol		Final Oil		Microemulsion (mL)	Position
	Oil (mL)	Slug (mL)	Oil (mL)	Slug (mL)		
0.0	4.55	4.55	0.00	3.50	5.60	Upper
1.0	4.55	4.55	3.10	4.50	1.50	Upper
2.0	4.55	4.55	1.00	4.30	3.80	Upper
3.0	4.55	4.55	3.50	4.50	1.10	Upper
4.0	4.50	4.50	2.96	2.94	3.10	Middle
5.0	4.55	4.55	3.76	3.74	1.60	Middle
6.0	4.55	4.55	4.50	3.53	1.05	Lower
7.0	4.50	4.50	4.30	1.48	3.20	Lower

**Table 3:** Measured properties of the core samples used in the experiments.

Cores	Length (cm)	Diameter (cm)	Pore Volume (cm^3^)	Porosity (%)	Permeability (mD)
1	15.24	3.81	34.55	20.00	183.00
2	15.08	3.79	32.91	19.35	125.90
3	15.23	3.79	33.63	19.44	96.30

**Table 4:** Nanoparticle/surfactant formulations and conditions used in the experiments.

Cores	Formulation	Pressure (psi)	Temperature (°C)	Injection Rate (cm^3^/min)	Oil API @ 23 °C
1	800 ppm DLCNP (functionalized) + 2% NaCl	1050	50	0.5	30
2	800 ppm DLCNP (Non-functionalized) + 0.5% APG + 2% NaCl	1050	50	0.5	30
3	800 ppm CNT + 0.5% APG + 2% NaCl	1050	50	0.5	30

**Table 5:** Quantitative elemental analysis from the XPS survey spectrum before and after carboxylic acid-functionalized carbon.

	Ball Milled (15 h) Carbon		Carboxylic Acid-Functionalized Carbon	
Element	Peak Binding Energy (eV)	Atomic %	Peak Binding Energy (eV)	Atomic %
C1s	285.00	74.43	285.09	66.86
O1s	531.93	20.40	532.03	29.74
Si2p	102.92	3.31	104.80	3.40
Ca2p	347.43	1.21	-	-
Mg1s	1304.86	0.66	-	-

**Table 6:** BET adsorption/desorption properties of the date carbon.

State of Carbon	BET Surface Area (m^2^/g)	Langmuir Surface Area (m^2^/g)	Average Pore Width (4V/A) (nm)	Total Volume (cm^3^/g)
Grinded carbon	254.37	338.53	2.10	0.13
Ball milled carbon (15 h)	331.32	446.98	3.67	0.30
Functionalized carbon	73.67	98.69	3.16	0.05

**Table 7:** Calculated phase behavior data of the APG—NaCl system.

Salinity NaCl (%)	Vol. of Oil V_o_ (mL)	Vol. of Surf. V_s_ (mL)	Vol. of Brine V_w_ (mL)	Solubilization of Oil (P_o_)	Solubilization of Brine (P_w_)
0	4.55	0.02	1.03	200	45.15
1	1.45	0.02	0.03	63.74	1.20
2	3.55	0.02	0.23	156.04	9.99
3	1.05	0.02	0.03	46.15	1.20
4	1.54	0.02	1.54	68.40	68.38
5	0.79	0.02	0.79	34.68	34.65
6	0.05	0.02	0.98	2.20	42.96
7	0.20	0.03	2.98	8.89	132.33

**Table 8:** Summary of the three EOR Formulations.

Expt. No.	Core No.	Formulations	Secondary Oil Recovery (%)	Tertiary Oil Recovery (%)	Total Oil Recovery (%)
1	1	Functionalized 800 mg/L (ppm) DLCNP and Distilled Water	40	9	49
2	2	Non-functionalized 800 mg/L (ppm) DLCNP, 0.5% APG 264 and 2% NaCl	44	45	89
3	3	800 mg/L (ppm) CNT, 0.5% APG and 2% NaCl	50	27	77

## Data Availability

Not applicable.

## Supplementary Materials

The following supporting information can be downloaded at: https://www.mdpi.com/article/10.3390/nano12081245/s1, Figure S1: Drop shape analyzer (DSA) 100, Figure S2: Core flooding experimental setup, Figure S3: BJH pore size distribution of the ground, ball-milled (15 h), and functionalized date carbon, Figure S4: Total oil recovery measured for the green surfactant and nanomaterial formulation, Figure S5: Total oil recovery measured for the three formulations tested, Figure S6: Comparison of the DLCNP with carbon nanotubes.

## Appendix A

**Table A1:** Ring dimensions.

Circumference of ring C, mm	60.1
$\frac{\mathrm{The}\;\mathrm{radius}\;\mathrm{of}\;\mathrm{the}\;\mathrm{ring}}{\mathrm{the}\;\mathrm{radius}\;\mathrm{of}\;\mathrm{the}\;\mathrm{wire}\;}=\frac{R}{r}$	53.8384846

**Table A2:** Density measurements at 25 °C.

Sample	H_2_O	Oil	100 ppm	200 ppm	400 ppm	600 ppm	800 ppm
***ρ*@ 25 °C g/cm^3^**	0.9970	0.8286	0.99718	0.99722	0.99725	0.99730	0.99735
Δρ **@ 25 °C g/cm^3^**	-	-	0.16858	0.16862	0.16865	0.16870	0.16875

Note: Δρ is the density difference between heavy and light phases.

**Table A3:** IFT and DLCNP values at different concentrations.

DLCNP Concentration (mg/L)	IFT (dyne/cm)
0	23.00
200	12.50
400	10.50
600	8.50
800	8.60

**Table A4:** IFT values for the DLCNP at different temperatures.

Temperature (°C)	IFT (dyne/cm)
25.5	13.91
35.0	14.76
45.0	15.79
81.3	17.09

**Table A5:** IFT values for the DLCNP at different pressures.

Pressure (psi)	IFT (dyne/cm)
3000	29.62
4000	30.33
5000	28.31
