# Supplementary material for: Date-Leaf Carbon Particles for Green Enhanced Oil Recovery
Source: Nanomaterials (Basel). 2022 Apr 7;12(8):1245. doi: 10.3390/nano12081245 (PMC9029107; doi:10.3390/nano12081245)
Supplement: Supplementary file 1 [file nanomaterials-12-01245-s001.zip › nanomaterials-1573754-supplementary.pdf]

Supporting Information

Date-Leaf Carbon Particles for Green Enhanced Oil Recovery

Bashirul Haq [1,*], Md. Abdul Aziz [2], Dhafer Al Shehri [1], Nasiru Salahu Muhammed [1], Shaik Inayath Basha [3], Abbas Saeed Hakeem [2], Mohammed Ameen Ahmed Qasem [2], Mohammed Lardhi [4] and Stefan Iglauer [5]

1 Department of Petroleum Engineering, King Fahd University of Petroleum and Minerals, Dhahran 31261, Saudi Arabia; alshehrida@kfupm.edu.sa (D.A.S.); g201907810@kfupm.edu.sa (N.S.M.)
2 Interdisciplinary Research Center for Hydrogen and Energy Storage, King Fahd University of Petroleum and Minerals, Dhahran 31261, Saudi Arabia; ashakeem@kfupm.edu.sa (A.S.H.); g200993710@kfupm.edu.sa (M.A.A.Q.)
3 Department of Civil and Environmental Engineering, King Fahd University of Petroleum and Minerals, Dhahran 31261, Saudi Arabia; g201407800@kfupm.edu.sa
4 Department of Reservoir Geoscience and Engineering, IFP School, 69 Avenue Paul Doumer, 92500 Rueil-Malmaison, France; mohammed.mohsen.lardhi@gmail.com
5 School of Engineering, Edith Cowan University, 270 Joondalup Drive, Joondalup, WA 6027, Australia; s.iglauer@ecu.edu.au
* Correspondence: bhaq@kfupm.edu.sa or bhaq225@gmail.com (B.H.); maziz@kfupm.edu.sa (M.A.A.)

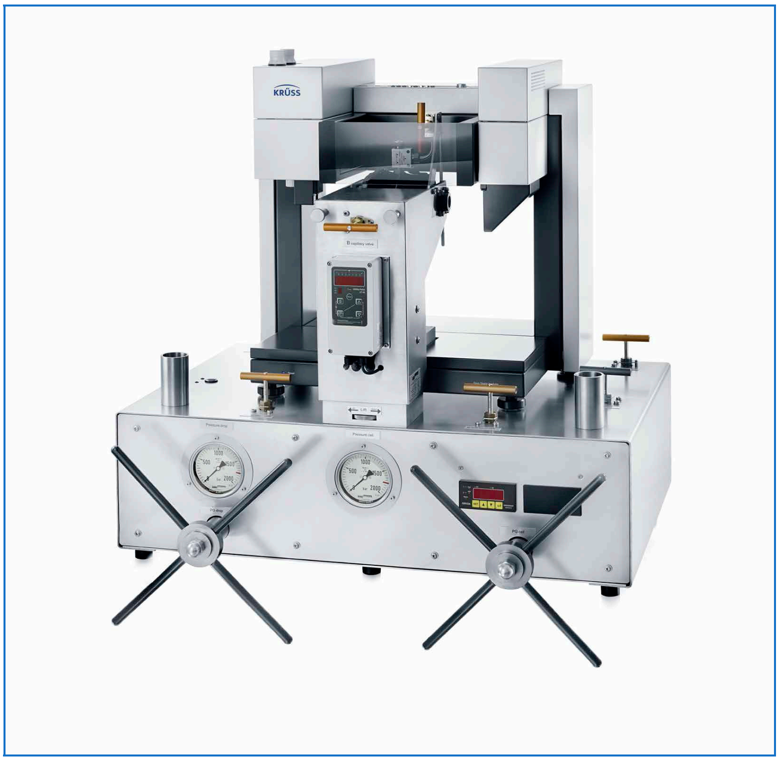

Figure S1. Drop shape analyzer (DSA) 100**.**

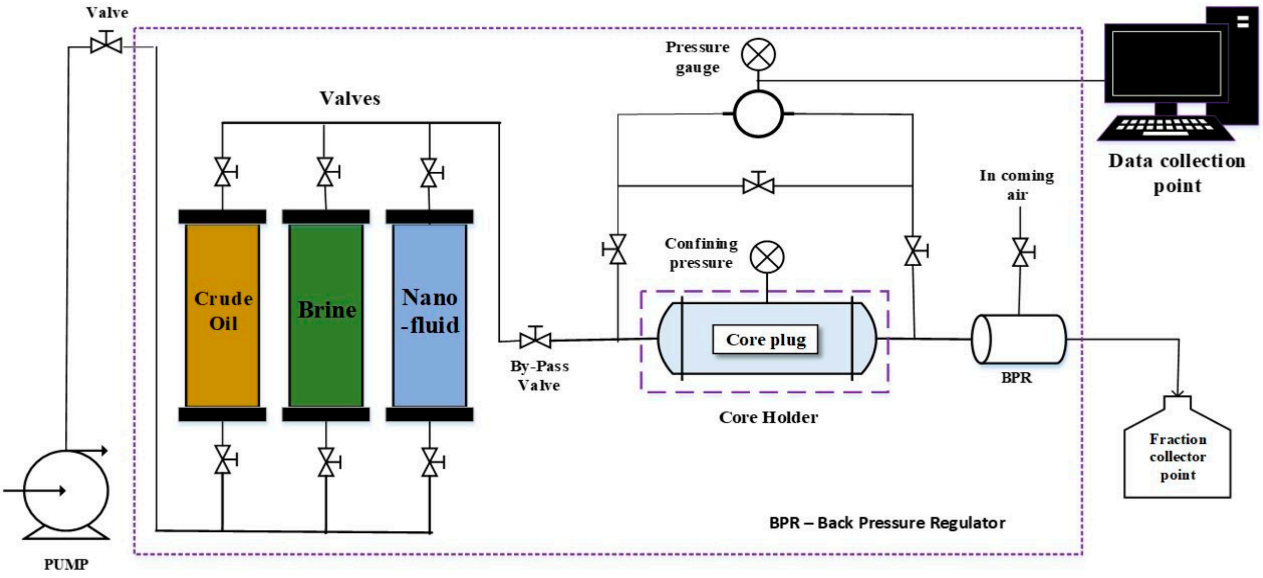

Figure S2. Core flooding experimental setup.

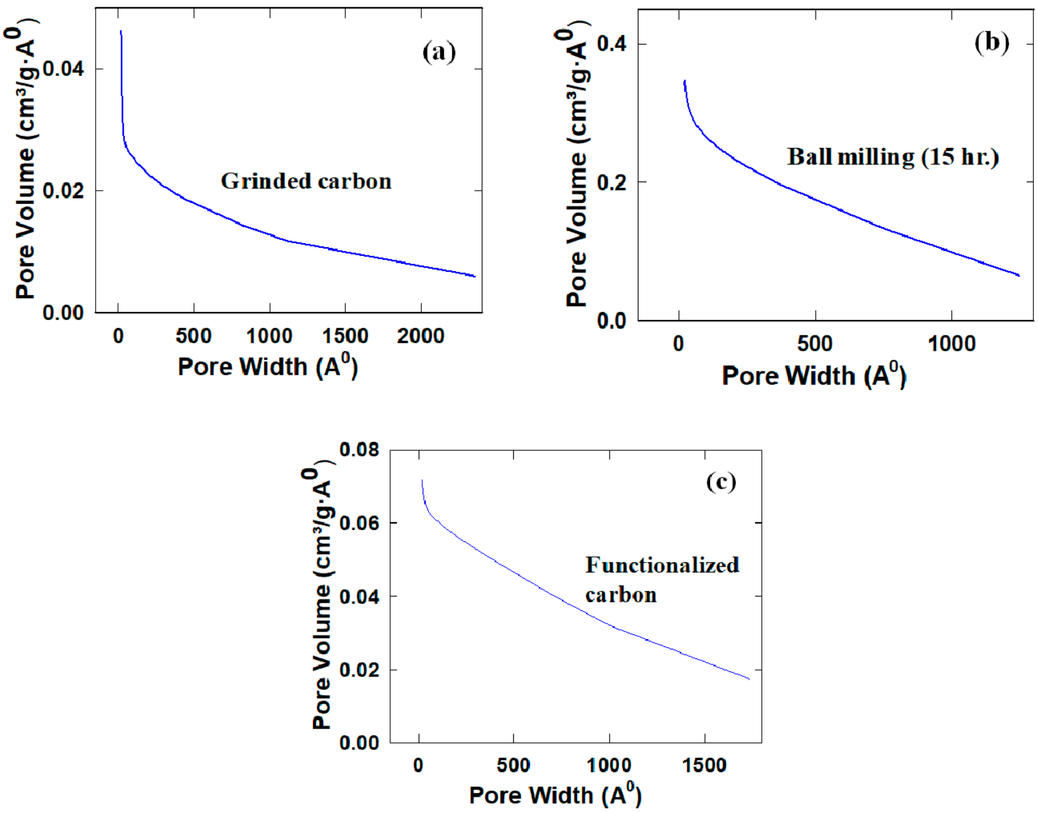

Figure S3. BJH pore size distribution of the (**a**) ground; (**b**) ball-milled (15 h; and (**c**) functionalized date carbon.

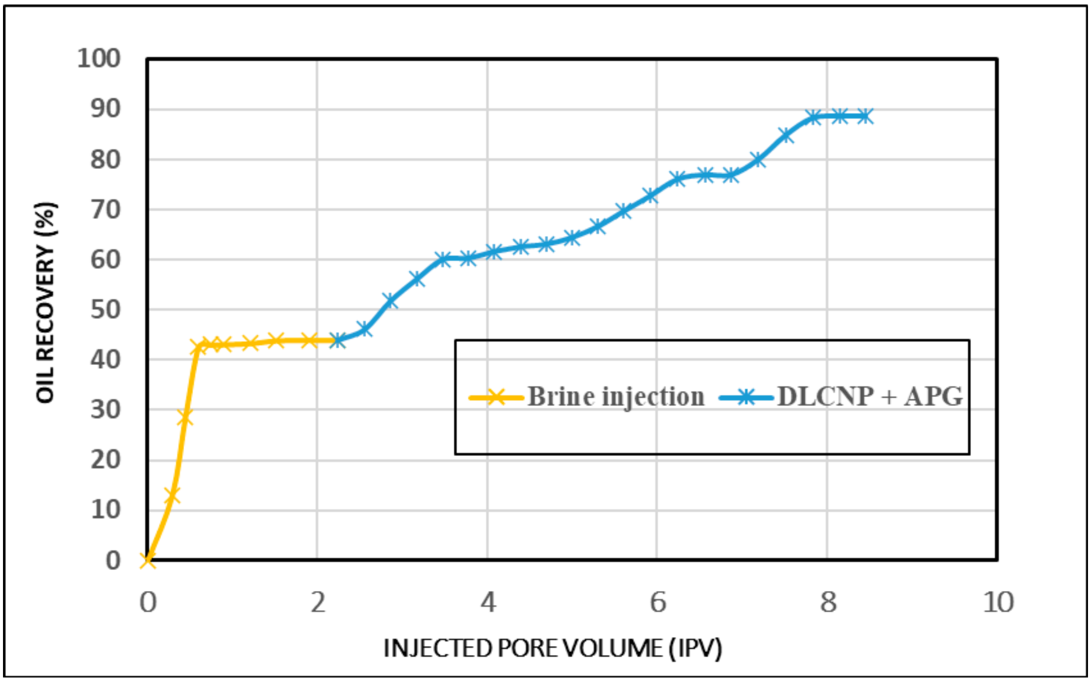

Figure S4. Total oil recovery measured for the green surfactant and nanomaterial formulation.

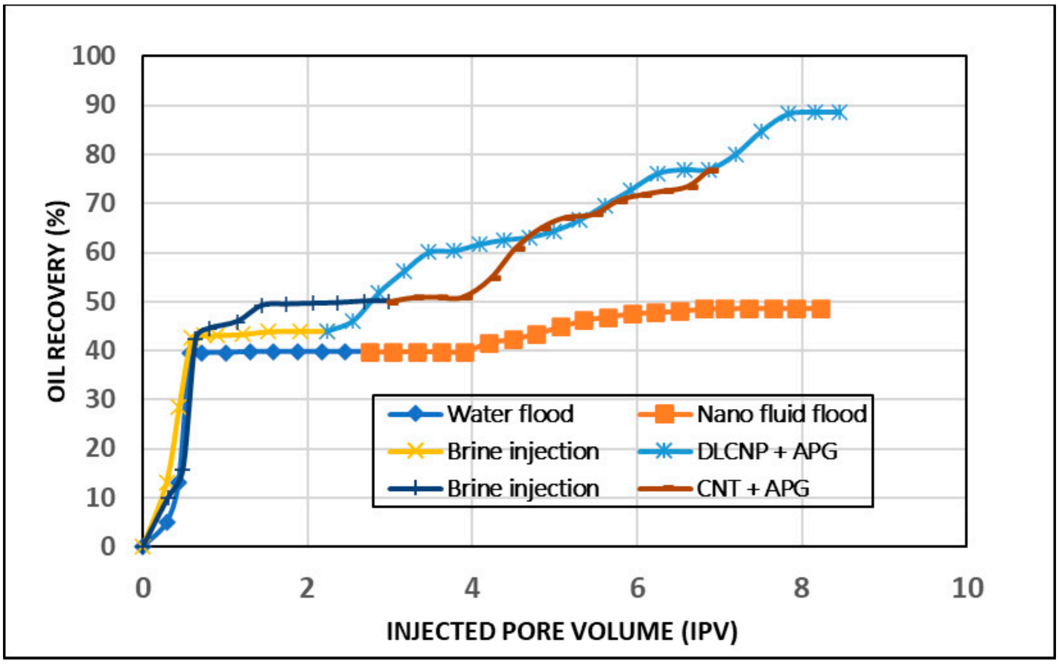

Figure S5. Total oil recovery measured for the three formulations tested.

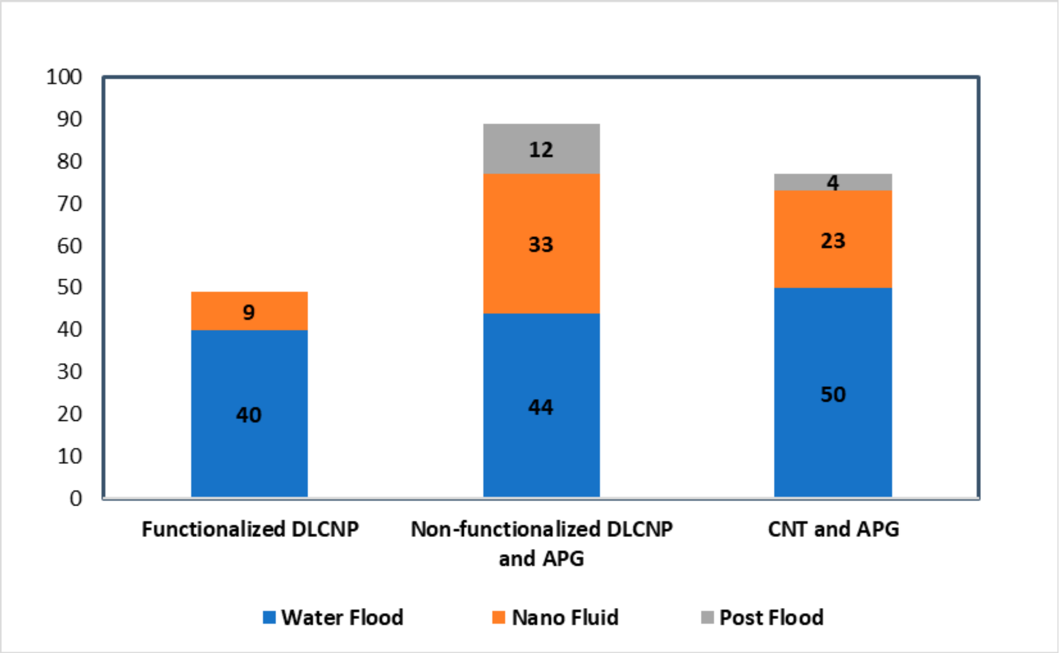

Figure S6. Comparison of the DLCNP with carbon nanotubes.
